# Can DALL-E 3 Reliably Generate 12-Lead ECGs and Teaching Illustrations?

**DOI:** 10.7759/cureus.52748

**Published:** 2024-01-22

**Authors:** Lingxuan Zhu, Weiming Mou, Keren Wu, Jian Zhang, Peng Luo

**Affiliations:** 1 Department of Oncology, Zhujiang Hospital of Southern Medical University, Guangzhou, CHN; 2 Department of Urology, Shanghai General Hospital, Shanghai Jiao Tong University School of Medicine, Shanghai, CHN

**Keywords:** medical education, 12-lead ecg, artificial intelligence in medicine, dall-e 3, chatgpt

## Abstract

The recent integration of the latest image generation model DALL-E 3 into ChatGPT allows text prompts to easily generate the corresponding images, enabling multimodal output from ChatGPT. We explored the feasibility of DALL-E 3 for drawing a 12-lead ECG and found that it can draw rudimentary 12-lead electrocardiograms (ECG) displaying some of the parameters, although the details are not completely accurate. We also explored DALL-E 3's capacity to create vivid illustrations for teaching resuscitation-related medical knowledge. DALL-E 3 produced accurate CPR illustrations emphasizing proper hand placement and technique. For ECG principles, it produced creative heart-shaped waveforms tying ECGs to the heart. With further training, DALL-E 3 shows promise to expand easy-to-understand visual medical teaching materials and ECG simulations for different disease states. In conclusion, DALL-E 3 has the potential to generate realistic 12-lead ECGs and teaching schematics, but expert validation is still needed.

## Introduction

DALL-E is an artificial intelligence image generation model developed by OpenAI that creates images based on user-given text descriptions [1]. The biggest leap in the latest release of DALL-E 3, apart from its significantly improved drawing skills, is its integration into ChatGPT. This means it reduces the complexity of prompt engineering and allows ChatGPT to generate prompts for image creation itself, enabling multimodal output from ChatGPT [2]. With this breakthrough, we explored whether DALL-E 3 could extend the material for clinical research and medical education by drawing 12-lead ECGs and schematic diagrams for teaching purposes. This technical report aims to explore the feasibility of DALL-E 3 for drawing 12-lead ECGs and the ability to generate teaching illustrations.

## Technical report

Creating a 12-lead ECG

Electrocardiography is a common technique used in cardiovascular clinical diagnosis and treatment, utilizing an electrocardiograph to record each cardiac cycle of the heart from the surface of the body to produce graphs of changes in electrical activity [3]. We told ChatGPT-4 that we needed DALL-E 3 to create a standard 12-lead ECG and input the prompt it generated into DALL-E 3 to create the images. The prompt generated by ChatGPT-4 contains settings for basic ECG parameters, specifying values for heart rate, P-wave, T-wave, P-R interval, QT interval, and QRS complex (see Supplementary Material).

**Figure 1:**
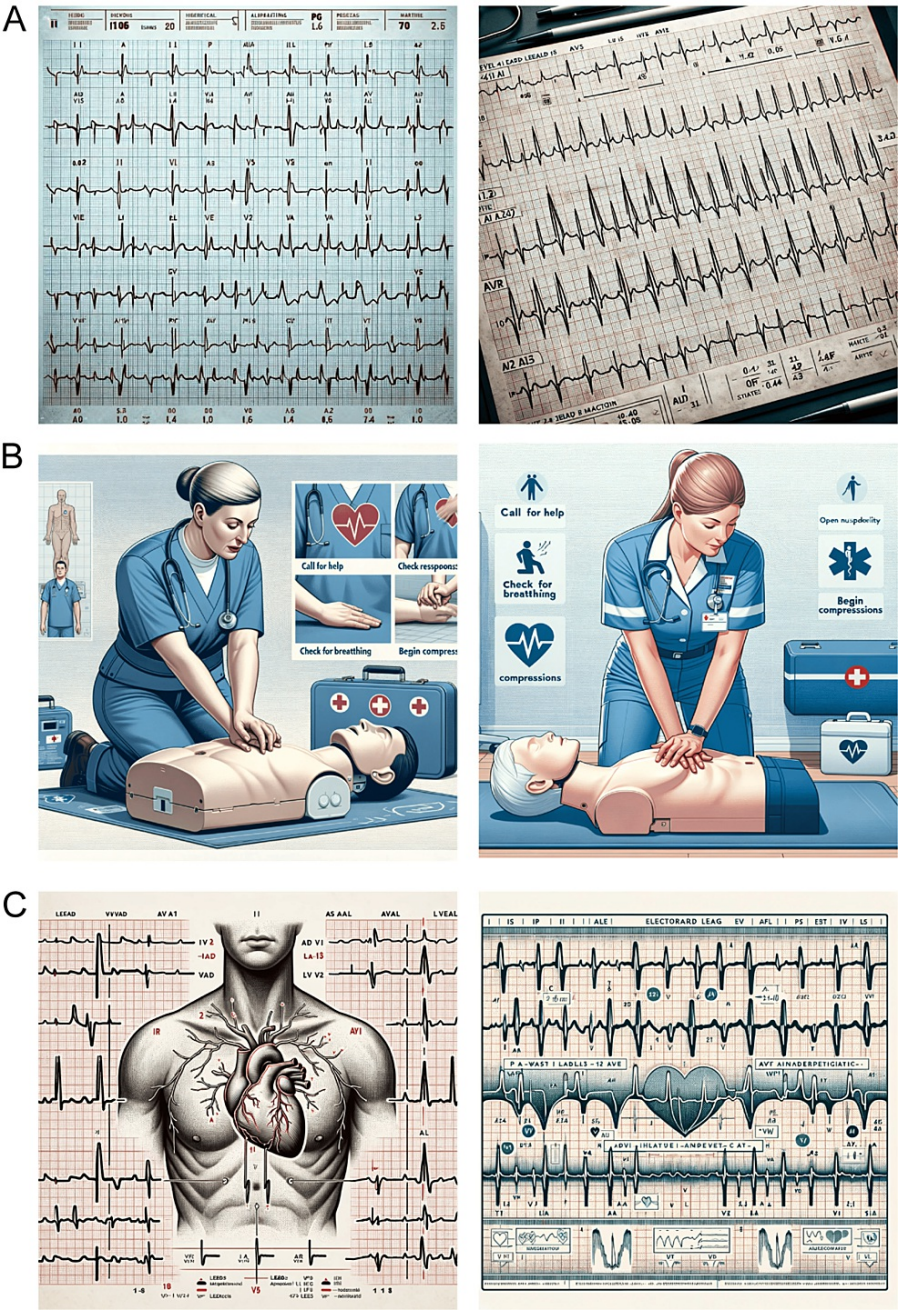
Images generated by DALL-E 3 (A) 12-lead ECG generated by DALL-E 3; (B) CPR instructional illustration generated by DALL-E 3; (C) ECG instructional illustration generated by DALL-E 3.

DALL-E 3 was able to generate an image containing visual features corresponding to some basic elements of ECG tracing, including P waves and QRS complexes (Figure 1A). However, the ECG it drew could not be considered a standard 12-lead ECG, as it failed to produce obvious T waves and contained several interfering waves. The heart rate reflected in the image was also incorrect. This ECG is more like an ECG image from a malfunctioning machine compared to an ECG from a normal person or patient, but the good thing is that there is a basic structure in place, although basic structures need further enhancement and improvement.

Creating a didactic illustration of resuscitation

*Illustration of CPR Techniques for Basic Life Support *(*BLS*)

High-quality illustrations can help students understand and memorize knowledge. CPR is a BLS knowledge [4,5]. We told ChatGPT-4 that we needed DALL-E 3 to create a didactic illustration of CPR techniques for BLS using the prompt it generated for us to enter into DALL-E 3 to create the image. The prompt generated by ChatGPT-4 proposed showing steps like calling for help, checking responsiveness, opening the airway, checking breathing, and including tools such as a defibrillator and first aid kit, accurately reflecting the content requirements for teaching CPR techniques.

The two CPR teaching illustrations drawn by DALL-E 3 were satisfactory, with the requirements in the prompt almost fully presented in the images. The emphasized steps were also shown separately in small images, and the core chest compression technique of CPR was very clearly illustrated with exquisite visual effects, combining practicality and vividness (Figure 1B).

Illustration for ECG Teaching

ECG is another BLS that should be mastered. We tried drawing ECG teaching illustrations. DALL-E 3 drew an image of waveforms by using love heart shapes and the human heart element, which was close to the principle of ECG and at the same time increased the fun and readability of the illustration, but it should be noted that the waveforms of the ECG in the illustration were not completely accurate (Figure 1C).

## Discussion

The results of this exploratory study demonstrate the potential of DALL-E 3 to generate basic medical illustrations and diagrams that could be useful for clinical research and medical education. However, there are also clear limitations that need to be addressed before DALL-E 3 illustrations can be reliably utilized.

For 12-lead ECG generation, while DALL-E 3 was able to produce an image containing some key elements of an ECG tracing, the waveform itself did not reflect a physiologically accurate or interpretable ECG. The heart rate was inaccurate, and important features like T waves were missing. This suggests that the AI does not yet have a robust enough understanding of ECG physiology and waveform characteristics to produce valid examples. Much more training data and prompt engineering would likely be needed to get clinically useful 12-lead ECG outputs.

The CPR teaching illustrations provided more promising results. DALL-E 3 successfully incorporated the key steps outlined in the prompt to produce clear, vivid illustrations of CPR techniques. This indicates that it can follow instructions well for depicting medical procedures. However, the two illustrations in Figure 1B contain some spelling errors, which are attributable to the inherent limitations of artificial intelligence image generation models including DALL-E 3. Regardless of these imperfections, the illustrations could still serve as helpful schematics for students learning CPR. However, it is also unclear if DALL-E 3 would perform as well for more complex procedural illustrations without appropriate training. There is also the risk of depicting subtle inaccuracies in techniques if not carefully reviewed by medical experts.

For the ECG teaching illustration, creativity was demonstrated in representing waveforms as heart shapes. However, key details of actual ECG wave morphology were missing or inaccurate. A review by cardiologists would be essential to ensure valid educational content.

In summary, while promising for simple medical illustrations, DALL-E 3 does not yet appear capable of generating complex, physiologically accurate diagrams and ECGs needed for clinical applications. Much more refinement of the technology, input prompts, and training data would be required with extensive expert validation needed for any utilization in medical education or patient care settings [6]. However, DALL-E 3 shows potential value for the future if limitations can be addressed through further AI development and validation. The next key steps could include assembling a dataset of >1000 expert-validated ECG tracings to further train parameters and enhance accuracy. Additionally, quantitative scoring of output quality, with iterative feedback-driven improvements, could also optimize fidelity.

## Conclusions

In conclusion, this exploratory study shows that DALL-E 3 has the potential for generating basic medical illustrations, as demonstrated through its ability to depict concise and vivid CPR instructional graphics based on ChatGPT-4 prompts. However, there are clear limitations regarding its capacity to produce physiologically accurate and clinically valid outputs like 12-lead ECG tracings. While able to draw rudimentary ECGs, DALL-E 3 did not reflect key elements of standard waveform morphology. Significant further refinement of the AI technology and training data, alongside extensive medical expert validation, would be imperative before DALL-E 3-generated content could be reliably utilized for clinical or educational purposes. However, the success in depicting core elements of medical procedures indicates there is potential for AI systems such as DALL-E 3 to assist in medical illustration and education if current limitations can be adequately addressed.

## Appendices

Supplementary Material. The prompts used to generate the images in this study.

Prompt related to Figure 1A: Create a realistic and detailed image of a twelve-lead electrocardiogram (ECG) similar to what would be seen on a medical report. Each of the twelve leads should be labeled accordingly (I, II, III, aVR, aVL, aVF, V1, V2, V3, V4, V5, V6). The heart rate should be depicted as 70 beats per minute, with the presence of P waves that are regular in direction. Each P wave duration should be 0.12 seconds, with a P-R interval of 0.15 seconds. The QRS complex should have a duration of 0.06 seconds, and the QT interval should be 0.4 seconds. Ensure that the QRS complex follows the P wave, and the T wave should be in the same direction as the main wave of the QRS complex.

Prompt related to Figure 1B: Create a detailed and educational illustration showcasing the steps of basic life support (BLS) for CPR. The image should depict a calm and professional environment with a rescuer performing CPR on a mannequin. The rescuer, a middle-aged Caucasian female in a blue medical uniform, should be shown in the correct hand placement for chest compressions. Include a clear view of the rescuer's arms straight and positioned above the mannequin's chest, demonstrating the compression technique. To the side, a text overlay should present step-by-step instructions: Call for help, Check responsiveness, Open the airway, Check for breathing, and Begin compressions. Visual aids, such as arrows to indicate motion and proper hand positioning, should be included to enhance understanding. The background should be simple, with a focus on the action, and possibly include a defibrillator and first aid kit in the background for context. Aim for a professional, clean, and educational tone suitable for a training manual.

Prompt related to Figure 1C: Create a highly detailed and realistic schematic illustration of a standard twelve-lead ECG (electrocardiogram) tracing, suitable for educational purposes, with labeled leads and cardiac waveforms. Include annotations for the P wave, QRS complex, T wave, and U wave where applicable. Each lead should clearly show the typical waveform morphology associated with that lead's view of the heart. The illustration should be clean, without any patient-specific abnormalities, to serve as a generic example for teaching the fundamentals of ECG interpretation. Please ensure the diagram has a professional and clinical appearance.
